# Human cord blood-derived platelet lysate enhances the therapeutic activity of adipose-derived mesenchymal stromal cells isolated from Crohn’s disease patients in a mouse model of colitis

**DOI:** 10.1186/s13287-015-0166-2

**Published:** 2015-09-09

**Authors:** Dorian Forte, Marilena Ciciarello, Maria Chiara Valerii, Luigia De Fazio, Elena Cavazza, Rosaria Giordano, Valentina Parazzi, Lorenza Lazzari, Silvio Laureti, Fernando Rizzello, Michele Cavo, Antonio Curti, Roberto M. Lemoli, Enzo Spisni, Lucia Catani

**Affiliations:** Institute of Hematology “L. & A. Seràgnoli”, Department of Experimental, Diagnostic and Specialty Medicine, Policlinico S. Orsola-Malpighi, University of Bologna, via G. Massarenti 9, 40138 Bologna, Italy; Department of Biological, Geological and Environmental Sciences, Biology Unit, University of Bologna, Via Selmi 3, 40126 Bologna, Italy; Cell Factory, Fondazione IRCCS Ca’ Granda Ospedale Maggiore Policlinico, via F. Sforza 35, 20122 Milano, Italy; Inflammatory Bowel Disease Unit, Policlinico S. Orsola-Malpighi, University of Bologna, Via Massarenti 9, 40138 Bologna, Italy; Chair of Hematology, Department of Internal and Specialty Medicine (DiMI), University of Genoa, Viale Benedetto XV 6, 16132 Genoa, Italy

## Abstract

**Introduction:**

Due to their immunomodulatory properties, mesenchymal stromal cells (MSCs) have been used for auto-immune disease treatment. Crohn disease (CD) and ulcerative colitis are two major inflammatory bowel diseases (IBDs), resulting from pathological immune responses to environmental or microbial antigens. Preclinical and clinical studies have suggested that MSC-based cellular therapy hold promising potential for IBD treatment. However, open issues include the selection of the proper cell dose, the source and the optimal route of administration of MSCs for more effective results. Platelet lysate has gained clinical interest due to its efficacy in accelerating wound healing. Thus, we propose to combine the administration of MSCs with a human umbilical cord blood-derived platelet lysate (hCBPL) as a novel strategy to improve MSC-based therapy for IBD resolution.

**Methods:**

Colitis was induced in 8-week-old C57BL/6J mice by daily oral administration of dextran sulphate sodium (DSS) (1.5 % w/v in tap water) for 9 days. MSCs were isolated from adipose tissue of CD patients (adCD-MSCs), expanded in proliferation medium, resuspended in hCBPL or PBS and administrated via enema for three times (1 × 10^6^ cells/mouse/time) every other day starting on day +7 from DSS induction. The colitis evolution was evaluated by daily monitoring of body weight, stool consistency and bleeding. Histopathological analysis was performed. Inflammatory cytokine plasma levels were determined. adCD-MSCs stained with lipophilic membrane dye Nile Red, were injected in DSS mice as described above. Colon section of mice sacrificed 24 hours after last cell administration, were analyzed by confocal microscopy.

**Results:**

We found that adCD-MSCs could be easily isolated and expanded from CD patients. Upon injection, adCD-MSCs exerted a therapeutic effect on DSS-induced colitis. Moreover, hCBPL increased adCD-MSCs efficacy by significantly reducing colitis scores, extension of the colon inflamed area and plasma levels of inflammatory mediators. Finally, Nile Red staining of MSCs is very efficient, stable and does not impair their vitality and function. Nile Red-labelling was clearly detected in the colitic area of adCD-MSCs injected mice and it was significantly brighter in the colon sections of mice that had received adCD-MSCs/hCBPL.

**Conclusions:**

In summary, with this study we propose a novel and promising adCD-MSC/hCBPL-based therapy for refractory IBDs.

**Electronic supplementary material:**

The online version of this article (doi:10.1186/s13287-015-0166-2) contains supplementary material, which is available to authorized users.

## Introduction

Mesenchymal stromal cells (MSCs) are multipotent adult stem cells [1] which can differentiate in vitro and in vivo into several tissue lineages originating from the three germinal layers [2, 3]. MSCs are reported to be immunoprivileged as well as immunosuppressive by inhibiting the activation, proliferation, and function of immune cells by different mechanisms of action [4–7]. These features make MSCs an increasingly attractive model for cellular therapy, bioengineering, and gene therapy [8]. Indeed, several preclinical studies have shown that MSCs can be used therapeutically to repair damaged tissues [9, 10]. Initially, MSCs were thought to mediate tissue and organ repair by virtue of their multilineage differentiation potential. However, it is now widely believed that in response to tissue injury, MSCs home to the site of damage to support tissue repair through the production of trophic factors, including growth factors, cytokines, and antioxidants, which also provide the basis for their capacity to modulate immune response [11]. In recent years, owing to their immunomodulatory properties, MSC transplantation has been used for the treatment of graft-versus-host disease (GVHD) following allogeneic stem cell transplantation, and several autoimmune diseases [12–16]. Inflammatory bowel disease (IBD), including Crohn's disease (CD) and ulcerative colitis (UC), is a chronic and relapsing autoimmune disorder. Although the etiology of IBD is not yet fully defined, it is generally agreed that a complex interplay between local immune reactions and environmental factors contribute, in genetically susceptible individuals, to disease initiation and progression [17]. Despite the advancement in IBD treatment, the current situation is still unsatisfactory. Indeed, poor results have been obtained in IBD patients with the use of anti-inflammatory drugs (e.g., tumor necrosis factor (TNF) inhibitors), which have been associated with poor and transient response. Furthermore, in 1/3 of patients, CD is complicated by perianal fistulas which rarely heal spontaneously or after medical treatment. Therefore, in most cases, surgical colon resection is still the ultimate alternative [18, 19]. Preclinical studies, performed using human MSCs in murine models [20–25], showed that MSCs are effective in ameliorating colitis severity by exerting an anti-inflammatory and/or immunosuppressive effect. Clinical trials demonstrated that autologous [26–30] or allogeneic [31] MSC transplantation is feasible and safe for the treatment of fistulas [26–30] or luminal disease [31] in patients with refractory CD. Although, the limited numbers of patients analyzed in each study do not allow conclusive demonstration of the effectiveness of the treatment, obtained results have suggested that MSC-based therapy holds promising potential for tissue regeneration and local anti-inflammatory effects in the treatment of IBD. Thus, in the attempt to improve the efficacy of MSC treatment, ongoing studies aim at evaluating the optimal source, the proper dose, and the route of MSC administration. MSCs can be isolated and expanded from several tissues including bone marrow (BM), adipose tissue, and umbilical cord blood (CB) [1]. Besides the BM, which is widely used in different experimental protocols and clinical trials and is considered a reference source, MSCs isolated from adipose tissue (ad-MSCs) have emerged as an attractive alternative for cell therapy, because of their prompt availability. ad-MSCs show proliferative capacity and differentiation potential similar to BM-derived MSCs [32]. Furthermore, immunosuppressive properties of ad-MSCs were comparable with [33], if not higher than [34, 35], those of BM-MSCs. ad-MSCs can efficiently control the GVHD in an in vivo mouse model [36], opening new perspectives for the clinical use of ad-MSCs in autoimmune diseases.

Platelet lysate has recently been proposed as substitute for fetal bovine serum (FBS) in MSC expansion for therapeutic purpose [37, 38]. Furthermore, platelet-rich plasma has gained clinical interest owing to its efficacy in accelerating wound healing and bone regeneration [39–42].

Here, we isolated MSCs from adipose tissue of CD patients (adCD-MSCs), and tested their efficacy in a murine model of dextran sulfate sodium (DSS)-induced experimental colitis (EC) [43]. We decided to combine the administration of adCD-MSCs with a human umbilical cord blood-derived platelet lysate (hCBPL). Indeed, we aimed to verify whether adCD-MSCs are effective in reducing the clinical signs of disease in a murine model of EC, and to test the administration of adCD-MSCs in combination with hCBPL as a novel strategy to improve MSC-based therapy for IBD resolution.

We found that adCD-MSCs can be easily isolated and expanded from CD patients and that hCBPL improved the efficacy of adCD-MSC-based treatment in ameliorating DSS-induced colitis.

## Materials and methods

### Isolation and expansion of ad-MSCs

After Ethics Committee approval (Independent Ethic Committee of the Azienda Ospedaliero-Universitaria Policlinico Sant’Orsola Malpighi of Bologna, approval number 1834/2013) and informed consent, ad-MSCs were obtained from four patients with CD, by surgical excision of healthy perifistula or subcutaneous area in sterile conditions or from the lower abdomen of four healthy female donors (as healthy donor controls), and processed as described previously [44]. Additional file 1 summarizes demographic data for experimental samples. Each patient gave written informed consent in accordance with the Declaration of Helsinki. Briefly, the adipose tissues were washed three times with phosphate saline buffer (PBS) and incubated with 1 % collagenase type 1 for 45 minutes at 37 °C. Mature adipocytes and undigested tissue fragments were separated from pellets of stromal vascular fraction (SVF) by centrifugation at 500 × *g* for 15 minutes. SVF cells were resuspended in culture medium (Dulbecco’s modified Eagle’s medium (DMEM) supplemented with 1 % penicillin/streptomycin, l-glutamine and 10 % FBS), plated and maintained in a humidified incubator at 37 °C and 5 % CO_2_. All nonadherent cells were removed after 24 hours. Medium was changed every 3–4 days until they reached 70–80 % confluence. Cells were then trypsinized (Lonza, Verviers, Belgium), replated at a density of 4000 cells/cm^2^, and used for experiments within passages 3–4. At the end of each passage (from passages 1 to 4) adCD-MSCs (*n* = 4) and MSCs from adipose tissue of healthy donors (adHD-MSCs; *n* = 4) were harvested and counted, and the population doubling (PD) rate was determined using the following formula:

$$ \mathrm{P}\mathrm{D}={ \log}_{10}\left(\mathrm{N}\right)/{ \log}_{10}(2) $$

where *N* is the ratio between the harvested cells and the seeded cells. The PD for each passage was calculated and added to the PD of the previous passages to generate data for cumulative PD.

### Immunophenotypical characterization of MSCs

For immunophenotype studies, dual-color immunofluorescence was performed using the following panel of phycoerythrin (PE)-conjugated or fluorescein isothiocyanate (FITC)-conjugated monoclonal antibodies: anti-human CD14, anti-human CD13, anti-human HLA-DR, anti-human CD34, and anti-human CD73 (BD Biosciences, Buccinasco, Italy); and anti-human CD105, anti-human CD44, anti-human CD45, anti-human CD90, and anti-human CD29 (Chemicon, Temecula, CA, USA). Negative controls were isotype-matched irrelevant monoclonal antibodies (BD Biosciences). MSCs (5 × 10^5^) were incubated in the dark for 15 minutes at 4 °C in PBS–1 % bovine serum albumin (BSA). Cells were rinsed in PBS and analyzed using a BD Accuri C6 flow cytometer (BD Biosciences). A minimum of 10,000 events was collected in list mode on FCS Express 4 Flow Research Edition Software (De Novo software, Glendale, CA, USA).

### Cell viability and proliferation assay

Trypan blue exclusion assay was used to determine the number of viable cells in each culture. Cell viability was determined by direct counting in a Burker chamber. Cells excluding the dye were counted as viable.

Cell proliferation was determined by CellTiter 96 AQueous One Solution Cell Proliferation Assay (Promega Italia, Milan, Italy). In brief, MSCs were seeded into a 96-well microplate at a density of 10,000 cells/cm^2^ 24 hours before the addition of the MTS solution (tetrazolium compound and phenazine methosulfate). Next, the cells were incubated for 1 hour at 37 °C in 5 % humidified CO_2_. Optical density value was measured using a microplate reader ELISA (Multiskan Ex; Thermo Electron Corporation, San Josè, CA, USA) at a wavelength of 492 nm. Each sample was measured in triplicate wells.

### MSC adipogenic and osteogenic differentiation

To induce adipogenic differentiation, adCD-MSCs were seeded at 2.1 × 10^4^ cells/cm^2^ in a Lab-Tek II coverglass chamber (Nalge-Nunc, Roskilde, Denmark) and grown for 3 days in Adipogenic induction medium (Lonza) containing additional human insulin, l-glutamine, (MCGS) Mesenchymal Cell Growth Serum, dexamethasone, indomethacin, 3-isobutyl-methyl-xanthine, penicillin/streptomycin followed by 3 days in adipogenic maintenance medium containing human insulin, l-glutamine, MCGS, penicillin/streptomycin. Both steps were repeated up to day 18 when cell cultures were stopped for histological staining. Fat droplets within adipocytes were identified using the Oil Red O staining method as described previously [45]. Briefly, cells were fixed in 10 % paraformaldehyde (PFA) in PBS for 1 hour at room temperature and rinsed in 60 % isopropanol. The isopropanol was then removed and the wells were completely dried and stained with a 0.6 % (w/v) Oil Red O solution (Sigma Aldrich, Saint Louis, MO, USA) for 15 minutes at room temperature with gentle shaking.

To induce osteogenic differentiation, adCD-MSCs were seeded at 3.1 × 10^3^ cells/cm^2^ and grown in Osteogenic Differentiation medium (Lonza) containing l-glutamine, MCGS, dexamethasone, ascorbate, β-glycerophosphate, penicillin/streptomycin. The media ware replaced every 3–4 days. Cell cultures were stopped at day 21 (3 weeks) for histological staining. Calcium deposition was determined using Alizarin red staining as described previously [45]. Briefly, cells were fixed in 10 % PFA in PBS for 15 minutes at room temperature, and then rinsed with PBS and distilled water. Fixed cultures were stained with 40 mM Alizarin red solution (Sigma Aldrich), pH 4.2, for 75 minutes at room temperature with gentle shaking and rinsed with distilled water.

### Nile Red MSC labeling

adCD-MSCs were stained with Nile Red, a lipophilic membrane dye (Sigma Aldrich), dissolved in dimethyl sulfoxide (DMSO; 0.4 mg/ml), and added to the medium at the final concentration of 0.5 % v/v. adCD-MSCs were washed in PBS and incubated with Nile Red for 15 hours at 37 °C in 5 % humidified CO_2_. After incubation, the cells were harvested at different time points (15, 24, 48, 72, and 120 hours), washed in PBS by centrifuging at 700 × *g* for 5 minutes, and analyzed. Nile Red fluorescence emission was measured by flow cytometry (FACSCanto™ II; BD Bioscences). The data analysis was carried out with the use of FACSDiva software (BD Biosciences, Buccinasco, Italy). For confocal microscopy analysis, adCD-MSCs (15,000/cm^2^) were incubated with Nile Red added to the culture medium at the final concentration of 0.5 % v/v, and grown on the coverslips for 15–72 hours. After incubation, MSCs were washed three times with PBS and fixed with 4 % PFA. After washing, cells slides were mounted in glycerol–PBS medium containing 50 mg/ml DABCO (1,4-diazabicyclo[2.2.2]octane). Confocal fluorescent images were obtained by a confocal microscope (LEICA TCS SP5; Leica Microsystems GmbH, Wetzlar, Germany) equipped with an argon/krypton ion laser. Nile Red was excited at 543 nm and the emission was collected in the spectral range from 650 to 700 nm. Optical sections were obtained at increments of 0.45 μm on the *z* axis and were digitized with a scanning mode format of 1024 × 1024 pixels. The image processing and quantitative evaluation of Nile Red intensity were performed using Leica TCS software (Leica Microsystems GmbH, Wetzlar, Germany). Negative controls consisted of samples not incubated with Nile Red. Fifteen images were analyzed for each tested condition.

### hCBPL preparation

CB units were collected from healthy newborns in plastic bags containing 29 ml citrate-phosphate-dextrose anticoagulant (Macopharma, Mouvaux, France) by trained midwives from vaginal deliveries and cesarean sections after informed written consent was obtained from the parents [46]. The Milano Cord Blood Bank is a public bank approved by the Health Regional Authority and certified for Cellular Therapy Product Collection, Processing, and Administration (Foundation for the Accreditation of Cellular Therapy- Joint Accreditation Committee-International Society for Cellular Therapy & European Group for Blood and Marrow Transplantation International Standards).

All of the CB collections were performed only after successful deliveries from healthy newborns with APGAR (Appearance, Pulse, Grimace, Activity, Respiration) scores between 9 and 10. CB units were processed within 24 ± 2 hours of collection to isolate platelets.

The CB units were centrifuged at 550 × *g* for 10 minutes to sediment red and white cells and to concentrate most of the platelets in the supernatant plasma. The resulting platelet fraction was then centrifuged at 2000 × *g* for 10 minutes to obtain a platelet pellet. This platelet fraction was diluted in an appropriate volume of supernatant (platelet-poor plasma) to a final concentration of 2 × 10^6^ platelets/μl. The platelets were then subjected to three cycles of freezing at −80 °C and thawing at 37 °C, and they were centrifuged at 2000 × *g* to produce the platelet lysate (patent procedure US 2011/0123503) [47].

### Animal maintenance

Eight-week-old male C57BL/6 mice were purchased from Charles River Laboratories (Lecco, Italy). Animals were housed in a controlled environment in collective cages, containing two mice each, at 22 ± 2 °C and 50 % humidity under a 12-hour light/dark cycle. Mice were allowed to acclimate to these conditions for at least 14 days before being randomized into experimental groups and had free access to food and water throughout the study. The experiments, approved by the institutional review board of the University of Bologna (approval number 22-78-2013), were performed according to Italian and European guidelines.

### Induction of experimental colitis and study design

Colitis was induced in C57BL/6J mice by oral administration of DSS (TdB Consultancy AB, Uppsala, Sweden) as described previously [43]. Briefly, DSS-treated mice received DSS in tap water (1.5 % DSS w/v). DSS–tap water was freshly prepared every day and administered to mice for 9 days and the average amount of DSS taken was recorded daily. adCD-MSCs were expanded in proliferation medium supplemented with 10 % FBS as described before, resuspended in hCBPL or PBS, and then administrated via enema three times (1 × 10^6^ cells/mouse/time) every other day starting on day +7 from DSS induction. This cell preparation protocol is compliant with the procedure we shall follow in the pending clinical trial. The animals were randomly assigned into the following groups of four mice each: CTR, mice received no treatment (control group); DSS+PBS, mice received DSS in tap water (1.5 %) for 9 days (on days 7, 9, and 11, mice received an enema administration of 150 μl PBS alone); DSS+adCD-MSCs, mice received DSS in tap water (1.5 %) for 9 days (on days 7, 9, and 11, mice received an enema administration of human adCD-MSCs (1 × 10^6^ cells/mouse/day) resuspended in 150 μl PBS); DSS+adCD-MSCs/hCBPL, mice received DSS in tap water (1.5 %) for 9 days (on days 7, 9, and 11, mice received an enema administration of adCD-MSCs (1 × 10^6^ cells/mouse/day) resuspended in 150 μl hCBPL); and DSS+hCBPL, mice received DSS in tap water (1.5 %) for 9 days (on days 7, 9, and 11, mice received an enema administration of 150 μl hCBPL). Before MSC administration, mice were mildly anesthetized using Zoletil-100 (10 mg/kg; Virbac, Carros, France), and Xilor (2.5 mg/kg; Bio98, Milan, Italy) by intramuscular injection. MSCs were administered via a 16G venous catheter (diameter 2 mm, length 48 mm; BD Bioscience, Buccinasco, Italy) that was advanced through the rectum into the colon, until the tip was 10 mm proximal to the anus. A venous catheter was applied to a 1 ml syringe and the MSC suspension was gently injected into the rectum. After MSC administration, mice were held by the tail in a vertical position for 30 seconds and monitored for at least 10 minutes, to ensure that the cell suspension was not ejected. The first set of experiments was carried out only with the first three experimental groups. The colitis evolution was monitored by daily recording of the body weight change of the animal study. In the second set of experiments, clinical assessment of inflammation was performed by daily monitoring of the general health condition, body weight, stool consistency, and bleeding. The Disease Activity Index (DAI) was evaluated daily by adding scores of stool consistency to scores of weight loss and occult blood and by averaging the scores of all mice in each group. Additional file 2 summarizes the criteria used to assign the scores. All experiments were repeated two or three times.

### Histopathological analysis

All mice were anesthetized as described before and sacrificed by cervical dislocation on day +18 or +22 (or day +12 in Nile Red-labeled cell experiments). The colon was excised, rinsed with saline solution, fixed in 10 % formalin, and embedded in paraffin wax or Optimal Cutting Temperature compound. Tissue sections (5 μm thick) were collected on coated slides and stained with hematoxylin and eosin (H&E). Histopathological scores were blindly determined as reported in Obermeier et al. [48]. Mice were scored individually. Ten sections for each animal were evaluated. Each group score represented the mean of at least four mice in each group. Additional file 3 summarizes the criteria used to assign histopathological damage scores. The histological damage score represents the sum of the epithelial damage and infiltration score, ranging from 0 (unaffected) to 8 (severe colitis).

### Cytokine measurements

Blood was collected from the caudal vein in sodium citrate-containing Eppendorf tubes. Plasma was obtained by centrifugation at 1000 × *g* for 15 minutes. Cytokine levels were determined using Luminex technology (BioPlex; Biorad, Segrate, Italy). The six-plexed pro mouse kit (interleukin (IL)-6, TNFα, IL-17, IL-1β, interferon gamma (IFNγ), IL-10;) was performed in 96-well filter plates following the manufacturer’s instructions as described previously [43]. The samples were analyzed in the Bioplex 200 instrument (Biorad, Segrate, Italy) and the concentration were estimated from a standard curve and expressed as pg/ml.

### Data analysis

Data are presented as mean ± SEM of at least three independent determinations. Statistical differences between groups were determined by one-way analysis of variance followed by Bonferroni’s post-hoc test for multiple comparison. All analyses were performed using GraphPad Prism software (version 6.0; La Jolla, CA, USA). *p* <0.05 was considered to indicate statistical significance.

## Results

### adCD-MSCs show typical MSC biological properties

**Fig. 1:**
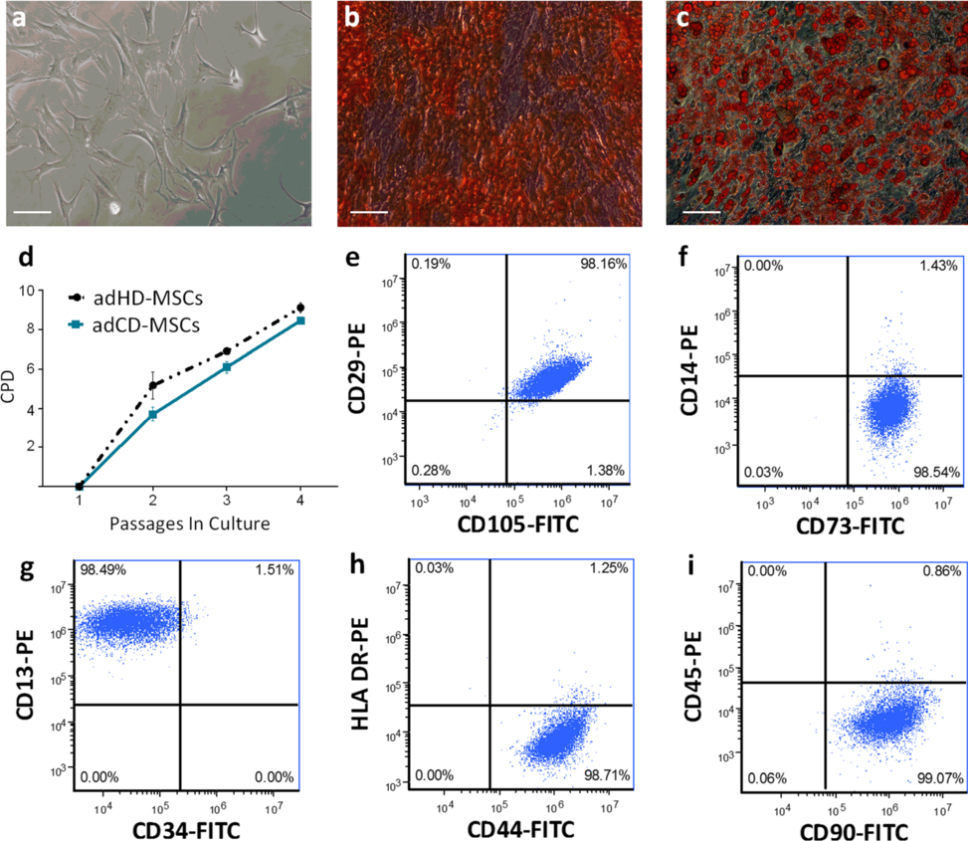
adCD-MSCs show typical MSC biological properties. **a** Representative field of exponential growing culture of adCD-MSCs. **b** Alizarin red staining of adCD-MSCs cultured for 3 weeks in osteogenic conditions and **c** Oil red O staining of adCD-MSCs cultured for 3 weeks in adipogenic conditions. Magnification 10×; scale bar, 100 μm. **d** Comparison of cumulative population doublings (*CPD*) calculated in adipose tissue-derived MSC cultures obtained from healthy donors (*adHD-MSC*) or adipose tissue-derived MSC cultures obtained from CD patients (*adCD-MSC*) at each passage. Results expressed as the mean ± SEM calculated from data obtained from four independent samples. Differences are not significant (*p* value not significant). **e–i** Flow cytometric immunophenotype of adCD-MSCs at passage 3. Representative dot plots. The analysis shows a predominant population of cells positive for CD90, CD29, CD73, CD105, CD13, and CD44, and negative for CD34, CD45, CD14, and HLA-DR. *FITC*, fluorescein isothiocyanate, *PE* phycoerythrin

We first isolated and expanded adCD-MSCs and characterized their biological properties. adCD-MSCs showed a typical spindle-shape morphology (Fig. 1a) and differentiation potential (Fig. 1b, c). We then measured the adCD-MSC proliferative capacity from isolation (passage 0) to passage 4 by calculating the PD rate. In the analyzed samples (*n* = 4), we found no significant differences as compared with MSCs derived from adipose tissue of healthy donors (adHD-MSCs, *n* = 4) (*p* value not significant) (Fig. 1d). Sample demographics are shown in Additional file 1. Regarding the immunophenotype, the majority of adCD-MSCs were, as expected, positive for CD73, CD13 (>98 %), CD105, CD90, CD44, and CD29 (>93 %), while they presented a low expression of HLA-DR (<2 %) and of hematopoietic markers such as CD34 (<2 %), CD45 (<1 %), and CD14 (<2 %) (Fig. 1e–i). A similar immunophenotype was observed in adHD-MSCs (data not shown). adCD-MSCs can thus be easily isolated and expanded from adipose tissue of CD patients and they show typical MSC biological properties according to the minimal criteria to define MSCs derived from adipose tissue [49].

### adCD-MSC therapy ameliorates colitis in a DSS mouse model of EC

**Fig. 2:**
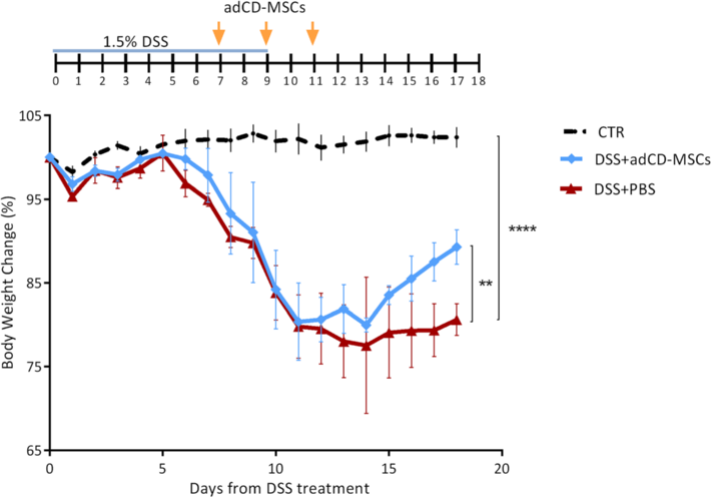
Treatment with adCD-MSCs protects against DSS-induced colitis. Mice received 1.5 % of DSS with their drinking water for 9 days. At days +7, +9, and +11 from DSS induction, mice are treated by enema with adCD-MSCs (1 × 10^6^ cells/mice/time) or phosphate saline buffer (*PBS*) alone. (*Top*) Schematic of the experiment. The evolution of colitis is monitored by body weight change expressed as a percentage of the initial body weight at day 0 (100 %). CTR, healthy control mice (*dotted black line*); DSS+adCD-MSCs, DSS-treated mice which received adCD-MSCs (*blue line*); DSS+PBS, DSS-treated mice which received PBS alone (*red line*). Data expressed as mean ± standard error of the mean (SEM). *n* = 4 mice per group. CTR vs. DSS+PBS, ****p <0.0001; DSS+adCD-MSCs vs. DSS+PBS, **p <0.01. *adCD-MSC* mesenchymal stromal cell isolated from adipose tissue of Crohn’s disease patients, *DSS* dextran sulfate sodium

**Fig. 3:**
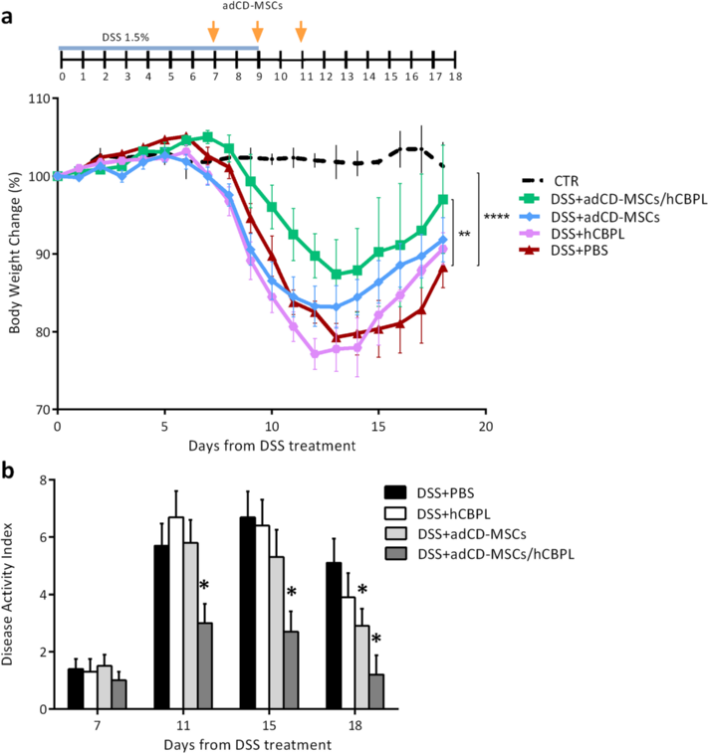
hCBPL improves the therapeutic efficacy of adCD-MSCs. Mice received 1.5 % of DSS with their drinking water for 9 days. At days +7, +9, and +11 from DSS induction, mice were treated by enema with adCD-MSCs (1 × 10^6^ cells/mice/time) with or without human umbilical cord blood-derived platelet lysate (*hCBPL*) or phosphate saline buffer (*PBS*) alone. Clinical evolution of colitis is monitored by body weight change and DAI evaluation. **a** Body weight changes during the course of the experiment are expressed as the percentage of the initial body weight at day +0. (*Top*) Schematic of the experiment. CTR, healthy control mice (*dotted black line*); DSS+adCD-MSCs, DSS-treated mice which received adCD-MSCs (*blue line*); DSS+adCD-MSCs/hCBPL, DSS-treated mice which received adCD-MSCs resuspended in hCBPL (*green line*); DSS+hCBPL, DSS-treated mice which received hCBPL alone (*pink line*); DSS+PBS, DSS-treated mice which received PBS (*red line*). Data expressed as mean ± SEM. *n* = 4–12 mice per group. CTR vs. DSS+PBS, ****p <0.0001; DSS+adCD-MSCs/hCBPL vs. DSS+PBS, **p <0.01; hCBPL vs. DSS+PBS, *p* value not significant. **b** DAI calculated by the combined score of weight loss, stool consistency, and bleeding, as detailed in Additional file 1. DAI is evaluated daily from day +7 (first administration of adCD-MSCs or adCD-MSCs/hCBPL or PBS or hCBPL) to day +18. Representative time points are shown. Data expressed as mean ± SEM. *n* = 4–12 mice per group. DSS+adCD-MSCs and DSS+adCD-MSCs/ hCBPL vs. DSS+PBS, *p <0.05. *adCD-MSC* mesenchymal stromal cell isolated from adipose tissue of Crohn’s disease patients, *DSS* dextran sulfate sodium

To test their potential therapeutic activity, adCD-MSCs were administrated by enema in a mouse model of DSS-induced EC, which we described recently [43]. In this model, mice presented initial and mild clinical signs of disease after the end of DSS treatment (day +9). The most evident clinical signs were recorded between days +9 and +15 [43], with a significant weight loss that peaked between days +11 and +14 [43] (see also Figs. 2 and 3). Thus, we decided to inject adCD-MSCs three times, every other day, starting from day +7 from DSS induction (Fig. 2). DSS+adCD-MSC-treated mice showed significantly reduced weight loss and faster body weight recovery compared with DSS+PBS-treated mice from day +11 (time of the last adCD-MSC administration) to the end of the observation, at each time point (Fig. 2) (*p* <0.01). adCD-MSCs are therefore effective in ameliorating DSS-induced colitis in our model of EC. Furthermore, we do not detect any side effect referring to the MSC administration by enema.

### hCBPL potentiates the effect of adCD-MSCs in an EC mouse model

Next, we investigated the activity of hCBPL to improve the efficacy of adCD-MSC treatment according to the same experimental design shown in Fig. 2. As reported in Fig. 3, the weight loss detected daily in the group of mice treated with DSS+adCD-MSCs/hCBPL was significantly lower than the weight loss of mice treated with DSS+PBS (*p* <0.01) or DSS+adCD-MSCs. Conversely, hCBPL alone in the absence of MSCs did not show any therapeutic effect (Fig. 3a).

Moreover, the DAI including clinical parameters of inflammation (weight loss, diarrhea, and rectal bleeding) [43] (see also Additional file 2) was daily recorded. DAI results (Fig. 3b, representative time points) confirmed the data obtained from the analysis of body weight change. In particular, in DSS+adCD-MSC/hCBPL-treated mice the DAI had already been significantly reduced at the end of MSC treatment (day +11) (*p* <0.05) and was further significantly reduced during the recovery phase (day +18), as compared with DSS+PBS-treated mice (*p* <0.05) (Fig. 3b). In DSS+hCBPL-treated mice, at each time point the DAI was not significantly different as compared with DSS+PBS-treated mice (*p* value not significant).

**Fig. 4:**
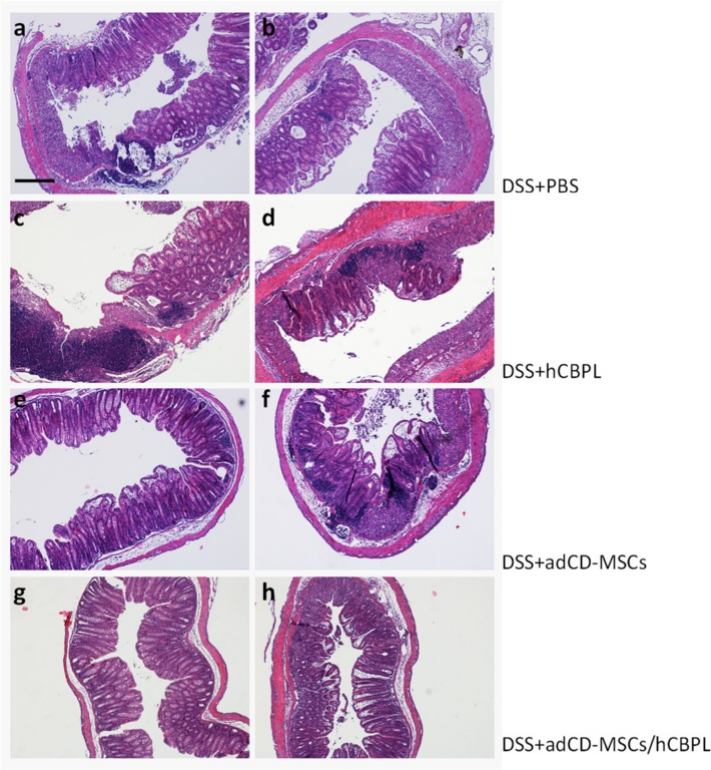
adCD-MSCs/hCBPL prevent DSS-induced pathology. Photomicrographs of H&E-stained paraffin sections of mouse colons treated as described before and harvested at day +22. Two representative examples are shown for each condition. Sections from **a, b** mice that received DSS, **c, d** mice that received DSS+hCBPL, **e, f** mice that received DSS+adCD-MSCs, and **g, h** mice that received adCD-MSCs/hCBPL. Magnification 50×; scale bar, 200 μm. *adCD-MSC* mesenchymal stromal cell isolated from adipose tissue of Crohn’s disease patients, *DSS* dextran sulfate sodium, *hCBPL* human umbilical cord blood-derived platelet lysate, *PBS* phosphate saline buffer

Histopathological examination of the colon sections in the distal and proximal tracts showed that adCD-MSC treatment significantly reduced the extension and the severity of the inflamed area and the infiltration of inflammatory cells (Fig. 4e, f), as compared with DSS-treated mice that received PBS only (Fig. 4a, b). Furthermore, DSS-treated mice that received adCD-MSCs/hCBPL showed only slight signs of colon inflammation and restored a normal appearance at day +22 (Fig. 4g, h). In contrast, as expected, DSS-treated mice that received hCBPL only showed a histopathological pattern similar to DSS+PBS-treated mice. A quantitative evaluation of histological damage was performed as described in Materials and methods and in Additional file 3. Histological score data (Table 1) confirmed that adCD-MSC treatment significantly reduced the epithelial damage and the infiltration of inflammatory cells as compared with DSS-treated mice that received PBS only. Furthermore, as expected from qualitative analysis, adCD-MSC/hCBPL-treated mice showed the lower histological score (*p* <0.05, compared with the DSS+PBS group) (Table 1). DSS-treated mice that received hCBPL only or PBS only showed a very similar histological score (*p* value not significant) (Table 1).

**Table 1:** Histological damage score: quantitative evaluation of histological damage in study mice

Mouse group	Mean ± SEM
DSS+PBS	7.10 ± 0.23
DSS+hCBPL	6.90 ± 0.28
DSS+adCD-MSCs	5.20 ± 0.29*
DSS+adCD-MSCs/hCBPL	4.70 ± 0.21*

Histological damage score was blindly determined from hematoxylin and eosin-stained paraffin sections of mouse colons harvested at day +22. Ten sections from each mouse and at least four mice for each group were evaluated. Data were presented as mean ± standard error of the mean (*SEM*). Histology was scored as indicated in Material and methods and in Additional file 3. Histological score represents the sum of the epithelial damage and infiltration score, ranging from 0 (unaffected) to 8 (severe colitis)

*Significant differences vs. DSS+PBS group, *p* <0.05

*adCD-MSC* mesenchymal stromal cell isolated from adipose tissue of Crohn’s disease patients, *DSS* dextran sulfate sodium, *hCBPL* human umbilical cord blood-derived platelet lysate, *PBS* phosphate saline buffer

**Fig. 5:**
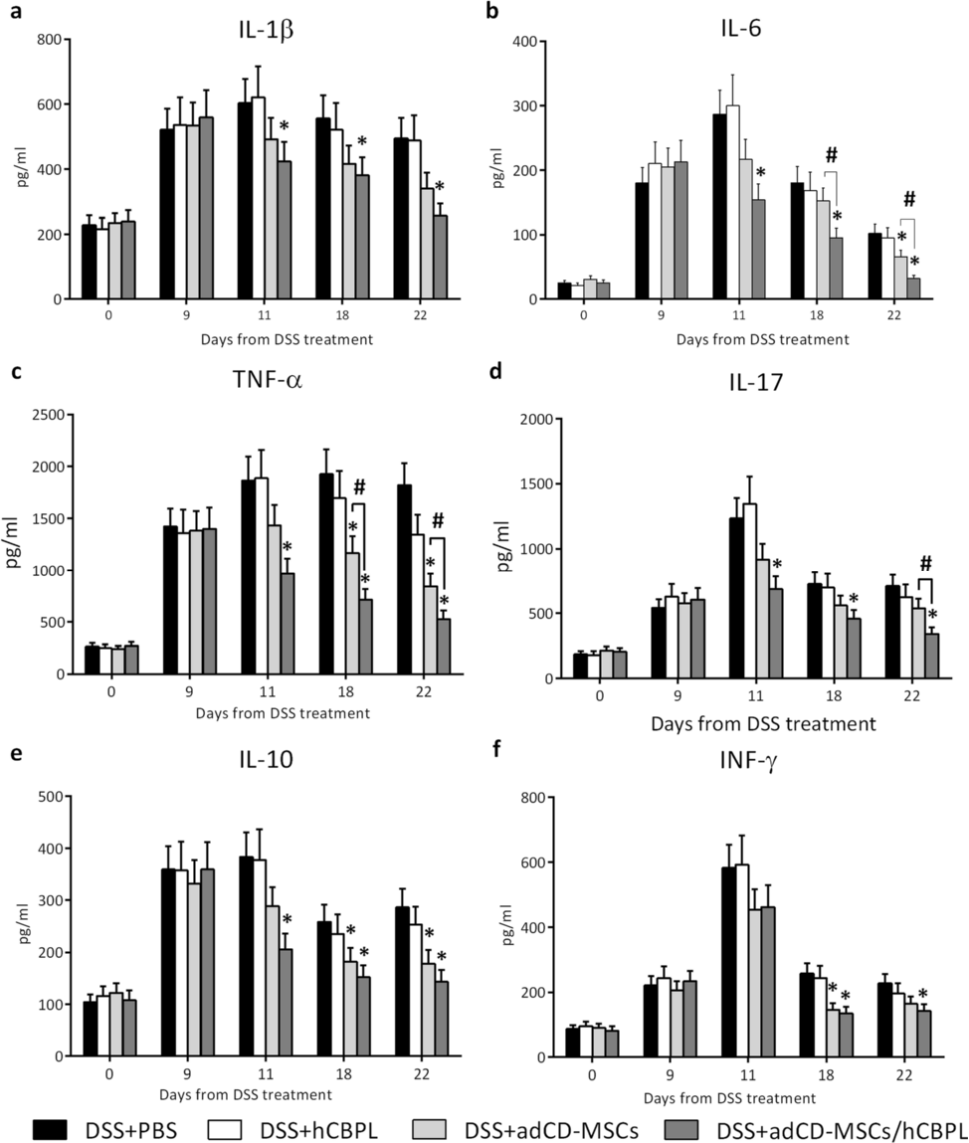
adCD-MSCs/hCBPL decrease systemic inflammatory response in DSS-induced colitis mouse model. Plasma was obtained from mice blood, collected from the caudal vein. **a–f** Cytokine levels (IL-1β, IL-17, TNFα, IL-6, IL-10, IFNγ) were measured using Bioplex assay at the start of the experiment (day +0), at the end of DSS treatment (day+9), after the administration of PBS (*black column*), hCBPL alone (*white column*), adCD-MSCs (*light gray column*), or adCD-MSCs/hCBPL (*dark gray column*) (day +11), at day +18, and at the end of the experiment (day +22). Data expressed as mean ± SEM. *n* = 4–8 mice per group. *p <0.05 with respect to DSS+PBS group; #*p* <0.05 in the comparison between the MSC groups. *adCD-MSC* mesenchymal stromal cell isolated from adipose tissue of Crohn’s disease patients, *DSS* dextran sulfate sodium, *hCBPL* human umbilical cord blood-derived platelet lysate, *IFN-γ* interferon gamma, *IL* interleukin, *PBS* phosphate saline buffer, *TNF*α tumor necrosis factor alpha

A putative mechanism of action for MSC therapy in autoimmune diseases is the downregulation of proinflammatory cytokines. Thus, we determined the plasma levels of IL-1β, IL-6, IL-17, IL-10, IFNγ, and TNFα in study mice. Notably, we found that adCD-MSCs reduced the systemic inflammatory responses in EC mice from day +11 to day +22 after DSS administration and their activity was significantly enhanced by hCBPL (Fig. 5). In particular, adCD-MSC/hCBPL administration significantly reduced the levels of all tested inflammatory cytokines, at days +18 and +22, if compared with cytokine levels in DSS-treated mice that received PBS only (*p* <0.05). Furthermore, the TNFα and IL-6 levels at days +18 and +22 (Fig. 5b, c) and the IL-17 level at day +22 (Fig. 5d) were decreased significantly in adCD-MSC/hCBPL-treated mice even with respect to adCD-MSC-treated mice (*p* <0.05) (Fig. 5). At each time point, the administration of hCBPL only did not cause significant changes in any cytokine levels compared with the administration of PBS only (*p* value not significant) (Fig. 5).

### Labeled adCD-MSCs/hCBPL localize in the injured tissue

**Fig. 6:**
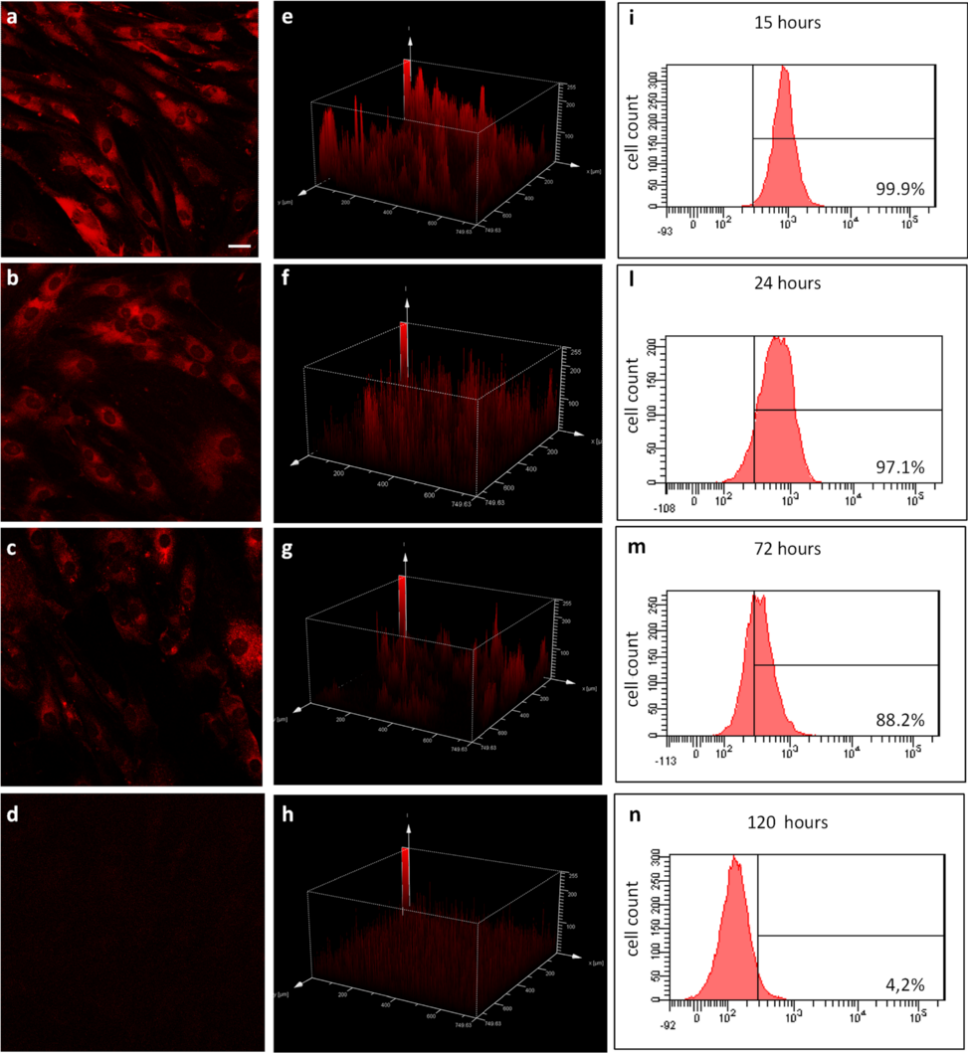
adCD-MSCs can be efficiently labeled by Nile Red staining. Fluorescence intensity and distribution of Nile Red staining. **a–d** Representative micrograph of stained cell cultures and **e–h** three-dimensional histograms of confocal analysis of fluorescence intensity (expressed as fluorescence intensity/pixel) for each time point. Fluorescence intensity of Nile Red-labeled adCD-MSCs analyzed by flow cytometry is high at **i** 15 hours, **l** 24 hours, and **m** 72 hours and **n** decreased significantly at 120 hours post labeling. Magnification 40×; scale bar, 20 μm

To better understand adCD-MSC trafficking, we set up a novel method for efficient cell labeling. We marked adCD-MSCs with Nile Red, a lipophilic membrane dye, commonly used for staining cellular lipid structures [50], and we analyzed labeled cells by confocal microscopy and flow cytometry. Nile Red-labeled cells are viable and maintained similar phenotypic, proliferative, and differentiative properties as compared with unlabeled cells (see Additional file 4). Furthermore, confocal images showed that adCD-MSCs were evenly stained by Nile Red. Flow cytometry analysis confirmed that Nile Red staining of adCD-MSCs was very efficient (99.9 % of positive cells 15 hours after the staining) and stable up to 72 hours. Fluorescence intensity of Nile Red-labeled adCD-MSCs, as indicated by both confocal microscopy and flow cytometry, significantly decreased at 120 hours post labeling (Fig. 6).

**Fig. 7:**
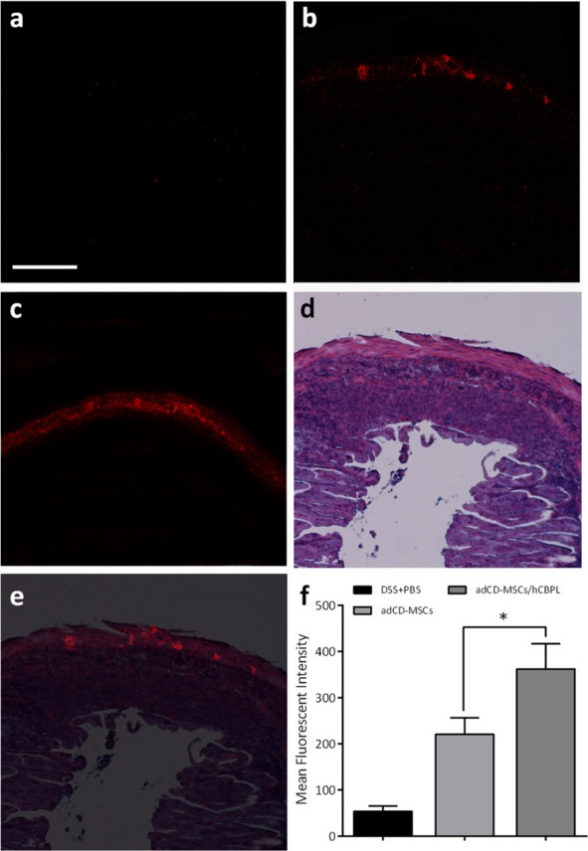
In vivo administration of Nile Red-labeled adCD-MSCs in DSS-treated colitic mice. DSS-treated mice were subjected to enema administration of Nile Red-stained adCD-MSCs. Colon tissue sections were collected 24 hours after the last MSC administration and were analyzed by confocal or optical microscopy. **a** DSS-treated mice colon sections show a low fluorescence background, in the absence of adCD-MSCs. Fluorescence signals in the presence of **b** Nile Red-stained adCD-MSCs or **c** adCD-MSCs/hCBPL. **d** Sections from consecutive segment were fixed and stained with H&E. **e** Merged images show that Nile Red fluorescence is present mainly in the muscular layers surrounding the colon injury. **f** Fluorescence quantification is presented as mean fluorescence intensity (± SEM). Data are representative of three mice for each experimental group and 15 acquired sections for each experimental condition. *p <0.05. Magnification 20×; scale bar, 150 μm. *adCD-MSC* mesenchymal stromal cell isolated from adipose tissue of Crohn’s disease patients, *DSS* dextran sulfate sodium, *hCBPL* human umbilical cord blood-derived platelet lysate, *PBS* phosphate saline buffer

Nile Red-labeled adCD-MSCs were injected by enema in DSS mice with the same schedule as the previous experiments. Injected mice were sacrificed 24 hours after last cell administration. Colon was then collected and tissue sections were analyzed by confocal microscopy. We found that a strong fluorescence was clearly visible in the tissue sections of adCD-MSC-injected mice (Fig. 7b), while a low fluorescence background was shown in DSS+PBS-treated mice (Fig. 7a). More importantly, the fluorescence signal in the colon sections of mice that received adCD-MSCs/hCBPL (Fig. 7c) was significantly brighter than in colon sections of mice administered adCD-MSCs alone, as confirmed by quantitative analysis of fluorescence intensity (*p* <0.05) (Fig. 7f). Merged images of sections from consecutive segment stained with H&E (Fig. 7d) showed that the fluorescence of Nile Red labeling was present mainly in the muscular ayers surrounding the colon injury (Fig. 7e). Overall, these data suggested that hCBPL stimulated the localization and/or transit of adCD-MSCs in the target tissue.

## Discussion

In spite of many recent improvements, current therapy for IBD is far from satisfactory [18, 19]. Thus, new and more effective therapeutic approaches are needed. In the present study, we proposed a novel strategy to improve the efficacy of MSC-based cellular therapy for IBD treatment. While previous reports demonstrated that human MSCs ameliorate both clinical and histopathological severity of EC [20–24], our study introduces innovative aspects aimed to increase MSC effectiveness. Indeed, we directly tested the efficacy of MSCs expanded from adipose tissue of CD patients in a murine model of EC. We found that adCD-MSCs can be easily isolated and expanded, maintaining their biological features. Our results demonstrated that adCD-MSCs are effective in reducing clinical and pathological signs of colitis. These findings are relevant in view of the use of autologous cells for the treatment of IBD patients.

Regarding in vivo study, there is no ideal animal model resembling human IBD, but certainly DSS-induced colitis is the most commonly used and has been validated as a proper model for the translation of mice data to human IBD. In our study we induced colitis using a reduced percentage of DSS (i.e., 1.5 %), converse to the murine model so far widely adopted (i.e., 3–5 % DSS). Thus, our model does not lead animals to death while presenting the peculiar changes in the colon structure, ulceration, dilatation, damage of the crypts, and inflammatory infiltrate (see Fig. 4a, b), but it is characterized by mild intestinal damage. This feature makes it very suitable and accurate for studying the dynamics of colitis during clinical remission and hence for evaluating therapeutic agent effects.

Here, we combined the administration of ad-MSCs with hCBPL. Usually these hemocomponents are obtained from platelets derived from peripheral blood. Recently, we developed a procedure to obtain a platelet product from umbilical CB [51]. CB platelets are able to release several growth factors contained in their granules such as platelet-derived growth factor PDGF-AB and PDGF-BB, vascular endothelial growth factor, hepatocyte growth factor, basic fibroblast growth factor, and transforming growth factor beta1 [47]. In addition, their trophic and immunomodulatory activities in animal models have already been tested by showing that CB platelets contain factors more likely to support soft tissue repair if compared with the peripheral blood counterpart [51]. Platelet lysate may also replace animal serum for the preparation of MSCs intended for clinical trials. However, previous studies demonstrated the proinflammatory activity of peripheral blood-derived platelet lysate (PBPL) [52]. Therefore, PBPL would not be an ideal addition for MSC preparation for cell therapy strategies in autoimmune disease. In our study, we used selected batches of FBS for MSC expansion to adhere as much as possible to the manufacturing protocol which will be used for the upcoming phase I trial. Indeed, the use of hCBPL is not feasible for large-scale MSC production for clinical trials. In the present paper, we showed that, also in an animal model of EC, hCBPL enhanced the regenerative effect of MSC-based treatment. adCD-MSCs clearly contribute to tissue repair as indicated by histopathological analysis (Fig. 4 and Table 1). We could speculate that, when coadministered with MSCs, hCBPL synergistically stimulates MSC effects. Furthermore, the activity of hCBPL in combination with ad-MSCs could explain the significant downregulation of T-helper type 1 cytokines such as IFNγ and TNFα, indicating that their beneficial effects rely, at least in part, on the anti-inflammatory mechanism. We also found that the circulating level of IL-10 followed the same trend of the other cytokines after MSC administration. Contrasting results regarding this issue have been reported recently. Specifically, some reports showed that local colonic anti-inflammatory cytokine IL-10 increased after MSC transplantation [20–22, 53]. Conversely, other studies showed that the IL-10 level remained unchanged after MSC administration in a DSS model but increased in a 2,4,6-trinitrobenzene sulfonic acid model [25]. Circulating IL-10 levels are not affected during the acute phase of DSS-induced colitis, but strongly increase during the chronic phase [54]. Since our DSS model induces mild colitis with a very short acute phase and a long chronic-like recovery phase, it is not surprising that MSC treatment—capable of ameliorating colitis, shortening the chronic phase, and accelerating the recovery—also reduces circulating IL-10 levels and/or probably impairs IL-10 accumulation. This suggests that, in our model, the MSC anti-inflammatory mechanism is probably IL-10 independent.

We then proposed a novel method for MSC labeling. For the first time, we employed Nile Red, a lipophilic membrane dye, commonly used for staining cellular lipid structures [50], to label viable MSCs and to track their presence/passage in the target tissue. Nile Red staining of MSCs is very efficient, is stable, and does not impair their vitality and function. Nile Red labeling was clearly detected in vivo after 24 hours from MSC transplantation in study mice. Moreover, the addition of hCBPL resulted in a significant increase in the amount of fluorescence signal detected in the colitic area. Notably, hCBPL showed greater potency than adult platelet-rich plasma preparations in stimulating MSC migration in a chemotaxis assay [55]. However, other mechanisms (i.e., stimulation of proliferation and/or increased survival of labeled cells in the injured area) may be involved. Furthermore, in vitro experiments showed that Nile Red-labeled adCD-MSCs were able to transfer lipophilic dye to unlabeled human colon mucosal epithelial cells but not vice versa (data not shown). The fluorescence we observed in the colon section of study mice thus relies on the presence and/or the transit of Nile Red-labeled adCD-MSCs in the site of injury, although we cannot rule out the hypothesis that the pattern is due to resident murine cells which received fluorescence by Nile Red-labeled adCD-MSCs.

Our results also demonstrated that MSCs can be administered safely and successfully by topical injection by enema. So far, murine [56–60] and human [20–25] MSCs have been injected in IBD murine models, intravenously or intraperitoneally. The topical injection allows the direct delivery of MSCs to the injured area where their beneficial effects are required. These findings are clinically relevant, especially in the perspective of using MSCs in those patients in whom CD is complicated by the development of fistulas, and are refractory to the current treatments.Recent phase I–II clinical studies suggested that local injection of MSCs in the submucosa in fistulizing lesions produces a considerable therapeutic benefit [27, 28, 30]. The small colon size in mice prevents the use of needles of a diameter sufficient to maintain the viability of the cells, and therefore submucosal injection of MSCs is not feasible in this model. However, the presence of Nile Red labeling in the muscular layers surrounding the colon injury after 24 hours from injection suggests the predictivity of our murine model.

## Conclusions

We isolated and expanded, ex vivo, and successfully injected by enema adCD-MSCs into DSS-treated mice. adCD-MSCs are effective in ameliorating the pathological and clinical features of DSS-induced colitis. hCBPL significantly improved adCD-MSC therapeutic effects. Local delivery of adCD-MSCs with hCBPL support may thus be considered a potentially safe and useful strategy for the treatment of IBD refractory to the current treatments.

## Additional files

Additional file 1: Table S1. Presenting sample demographics. Summary of the main features of CD patients from which adCD-MSCs were isolated. Data for healthy donors from which adHD-MSCs were isolated for the comparison of biological properties are also shown. (DOC 34 kb) Additional file 2: Table S2. Presenting DAI score criteria. Summary of the criteria used to assign the scores added to determine the DAI in mice daily. (DOCX 13 kb) Additional file 3: Table S3. Presenting histological damage score criteria. Summary of the criteria used to assign the scores added to determine the histological damage score. (DOC 31 kb) Additional file 4: Figure S1. Showing that Nile Red-labeled adCD-MSCs are viable and maintain phenotypic and differentiative properties as compared with unlabelled cells. Proliferation rate (cell titer assay), immunophenotype (flow cytometry), and differentiation potential (Alizarin red and Oil red O staining) of Nile Red-labeled adCD-MSCs (adCD-MSCs+NR) compared with unlabeled adCD-MSCs. Nile Red-labeled adCD-MSCs are viable and maintain phenotypic and differentiative properties compared with unlabeled cells. **a** Proliferation rate is evaluated by cell titer assay and expressed as fold-change taking the value of unlabeled cells at each time point as 1. Data expressed as mean ± SEM of at least three experiments. All differences are not significant (*p* value not significant). *NR* Nile Red. **b** Flow cytometric immunophenotype of Nile Red-labeled adCD-MSCs (adCD-MSCs+NR) is similar to that of unlabeled adCD-MSCs. Data expressed as mean ± SEM of at least two experiments. All differences are not significant (*p* value not significant). As a proof of differentiation potential, osteogenic and adipogenic process are shown. Alizarin red and Oil red O staining of unlabeled **c, d** and Nile Red-labeled **e, f** adCD-MSCs cultured for 3 weeks in osteogenic **c, e** and adipogenic conditions **d, f**. Magnification 20×; scale bar, 100 μm. (PDF 1843 kb)

## Abbreviations

adCD-MSC: Mesenchymal stromal cell isolated from adipose tissue of Crohn’s disease patients
adHD-MSC: Mesenchymal stromal cell derived from adipose tissue of healthy donors
ad-MSC: Mesenchymal stromal cell isolated from adipose tissue
BM: Bone marrow
BSA: Bovine serum albumin
CB: Cord blood
CD: Crohn’s disease
DAI: Disease Activity Index
DMEM: Dulbecco’s modified Eagle’s medium
DMSO: Dimethyl sulfoxide
DSS: Dextran sulfate sodium
EC: Experimental colitis
FBS: Fetal bovine serum
FITC: Fluorescein isothiocyanate
GVHD: Graft-versus-host disease
H&E: Hematoxylin and eosin
hCBPL: Human umbilical cord blood-derived platelet lysate
IBD: Inflammatory bowel disease
MSC: Mesenchymal stromal cell
PBPL: Peripheral blood-derived platelet lysate
PBS: Phosphate saline buffer
PE: Phycoerythrin
PFA: Paraformaldehyde
SEM: Standard error of the mean
SVF: Stromal vascular fraction
TNF: Tumor necrosis factor
UC: Ulcerative colitis
