# Supplementary material for: Human cord blood-derived platelet lysate enhances the therapeutic activity of adipose-derived mesenchymal stromal cells isolated from Crohn’s disease patients in a mouse model of colitis
Source: Stem Cell Res Ther. 2015 Sep 9;6(1):170. doi: 10.1186/s13287-015-0166-2 (PMC4564981; doi:10.1186/s13287-015-0166-2)
Supplement: Additional file 1: Table S1. — Presenting sample demographics. Summary of the main features of CD patients from which adCD-MSCs were isolated. Data for healthy donors from which adHD-MSCs were isolated for the comparison of biological properties are also shown. (DOC 34 kb) [file 13287_2015_166_MOESM1_ESM.doc]

**Human Cord Blood-derived Platelet Lysate Enhances The Therapeutic Activity Of Adipose Derived Mesenchymal Stromal Cells Isolated From Crohn Disease Patients In A Mouse Model Of Colitis**

Dorian Forte, Marilena Ciciarello, Maria Chiara Valerii, Luigia De Fazio, Elena Cavazza, Rosaria Giordano, Valentina Parazzi, Lorenza Lazzari, Silvio Laureti, Fernando Rizzello, Michele Cavo, Antonio Curti, Roberto M. Lemoli, Enzo Spisni and Lucia Catani

**Table S1. Sample Demographics**

| **CD patients** | | | | |
| --- | --- | --- | --- | --- |
| **Patient code** | **Age (years)** | **Sex** | **Distribution of disease** | **adipose tissue (gr)** |
| #CD1 | 32 | M | Crohn’s colitis | 36.9 |
| #CD2 | 55 | F | Crohn’s ileocolitis+perianal disease | 28.7 |
| #CD3 | 24 | F | Crohn’s proctitis | 52.2 |
| #CD4 | 27 | M | Crohn’s ileocolitis+perianal disease | 48.4 |
| **Healthy Donors** | | | | |
| **Patient code** | **Age (years)** | **Sex** | **Distribution of disease** | **adipose tissue (gr)** |
| #HD1 | 25 | F | absent | 20.5 |
| #HD2 | 47 | F | absent | 22.3 |
| #HD3 | 43 | F | absent | 22 |
| #HD4 | 40 | F | absent | 15 |
