# Supplementary material for: Human cord blood-derived platelet lysate enhances the therapeutic activity of adipose-derived mesenchymal stromal cells isolated from Crohn’s disease patients in a mouse model of colitis
Source: Stem Cell Res Ther. 2015 Sep 9;6(1):170. doi: 10.1186/s13287-015-0166-2 (PMC4564981; doi:10.1186/s13287-015-0166-2)
Supplement: Additional file 2: Table S2. — Presenting DAI score criteria. Summary of the criteria used to assign the scores added to determine the DAI in mice daily. (DOCX 13 kb) [file 13287_2015_166_MOESM2_ESM.docx]

**Human Cord Blood-derived Platelet Lysate Enhances The Therapeutic Activity Of Adipose Derived Mesenchymal Stromal Cells Isolated From Crohn Disease Patients In A Mouse Model Of Colitis**

Dorian Forte, Marilena Ciciarello, Maria Chiara Valerii, Luigia De Fazio, Elena Cavazza, Rosaria Giordano, Valentina Parazzi, Lorenza Lazzari, Silvio Laureti, Fernando Rizzello, Michele Cavo, Antonio Curti, Roberto M. Lemoli, Enzo Spisni and Lucia Catani

**Table S2. Disease activity index (DAI) score criteria**

| **Stool consistency** | **Bleeding** | **Weight loss** |
| --- | --- | --- |
| 0 = formed | 0 = normal color stool | 0 = no weight loss |
|  |  |  |
| 1 = mild-soft | 1 = brown color | 1 = 5-10% weight loss |
|  |  |  |
| 2 = very soft | 2 = reddish color | 2 = 11-15% weight loss |
|  |  |  |
| 3 = watery stool | 3 = bloody stool | 3 = 16-20% weight loss |
|  |  |  |
|  |  | 4 = >20% weight loss |

Summary of parameter and the criteria used to assign the scores added to daily determine Disease Activity Index (DAI) in mice study.
