# Supplementary material for: Human cord blood-derived platelet lysate enhances the therapeutic activity of adipose-derived mesenchymal stromal cells isolated from Crohn’s disease patients in a mouse model of colitis
Source: Stem Cell Res Ther. 2015 Sep 9;6(1):170. doi: 10.1186/s13287-015-0166-2 (PMC4564981; doi:10.1186/s13287-015-0166-2)
Supplement: Additional file 3: Table S3. — Presenting histological damage score criteria. Summary of the criteria used to assign the scores added to determine the histological damage score. (DOC 31 kb) [file 13287_2015_166_MOESM3_ESM.doc]

**Human Cord Blood-derived Platelet Lysate Enhances The Therapeutic Activity Of Adipose Derived Mesenchymal Stromal Cells Isolated From Crohn Disease Patients In A Mouse Model Of Colitis**

Dorian Forte, Marilena Ciciarello, Maria Chiara Valerii, Luigia De Fazio, Elena Cavazza, Rosaria Giordano, Valentina Parazzi, Lorenza Lazzari, Silvio Laureti, Fernando Rizzello, Michele Cavo, Antonio Curti, Roberto M. Lemoli, Enzo Spisni and Lucia Catani

**Table S3. Hystological damage score criteria**

| **Epithelial damage** | **Infiltration** |
| --- | --- |
| 0 = none | 0 = none |
|  |  |
| 1 = minimal loss of globelet cells | 1 = infiltrate around crypt bases |
|  |  |
| 2 = extensive loss of globelet cells | 2 = infiltrate in muscularis mucosa |
|  |  |
| 3 = minimal loss of crypts and extensive loss of globelet cells | 3 = infiltrate in muscularis mucosa with edema |
|  |  |
| 4 **=** extensive loss of crypts | 4 = infiltration of submucosa |

Summary of the parameter and the criteria used to assign the scores added to determine histological damage score. Histological damage score was blindly determined from hematoxylin-eosin (H&E)-stained paraffin sections of mouse colons harvested at day +22. Histological damage score represents the sum of the epithelial damage and infiltration score, ranging from 0 (unaffected) to 8 (severe colitis).
