# Supplementary figures and images for: Human cord blood-derived platelet lysate enhances the therapeutic activity of adipose-derived mesenchymal stromal cells isolated from Crohn’s disease patients in a mouse model of colitis
Source: Stem Cell Res Ther. 2015 Sep 9;6(1):170. doi: 10.1186/s13287-015-0166-2 (PMC4564981; doi:10.1186/s13287-015-0166-2)

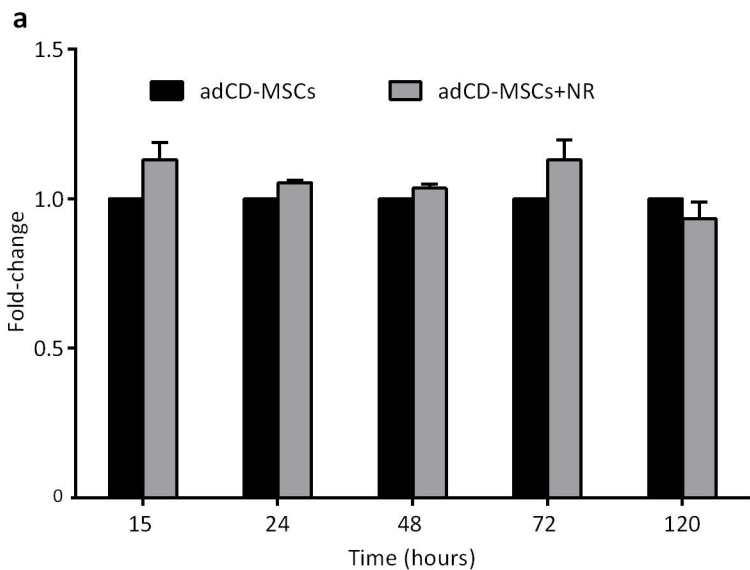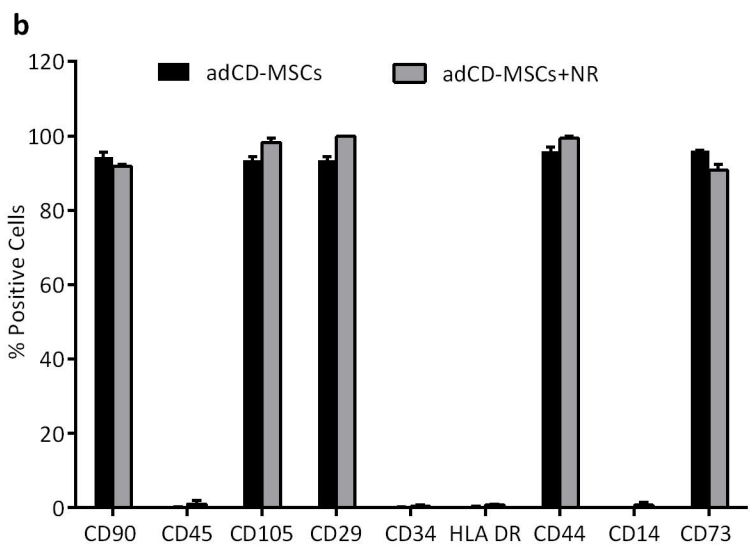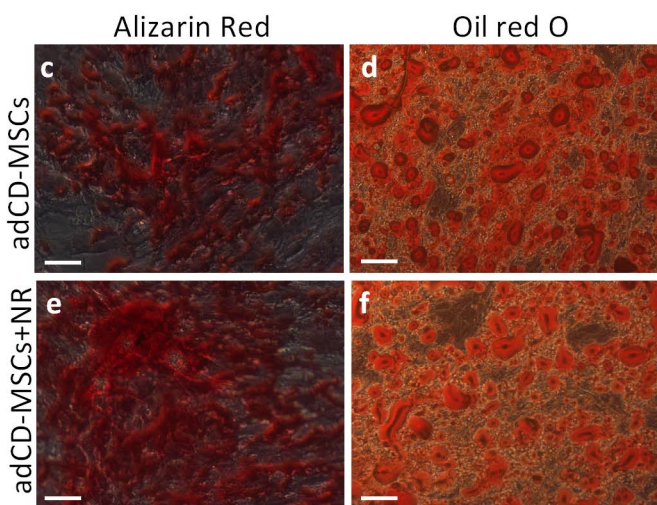

Supplement: Additional file 4: Figure S1. — Showing that Nile Red-labeled adCD-MSCs are viable and maintain phenotypic and differentiative properties as compared with unlabelled cells. Proliferation rate (cell titer assay), immunophenotype (flow cytometry), and differentiation potential (Alizarin red and Oil red O staining) of Nile Red-labeled adCD-MSCs (adCD-MSCs+NR) compared with unlabeled adCD-MSCs. Nile Red-labeled adCD-MSCs are viable and maintain phenotypic and differentiative properties compared with unlabeled cells. a Proliferation rate is evaluated by cell titer assay and expressed as fold-change taking the value of unlabeled cells at each time point as 1. Data expressed as mean ± SEM of at least three experiments. All differences are not significant (p value not significant). NR Nile Red. b Flow cytometric immunophenotype of Nile Red-labeled adCD-MSCs (adCD-MSCs+NR) is similar to that of unlabeled adCD-MSCs. Data expressed as mean ± SEM of at least two experiments. All differences are not significant (p value not significant). As a proof of differentiation potential, osteogenic and adipogenic process are shown. Alizarin red and Oil red O staining of unlabeled c, d and Nile Red-labeled e, f adCD-MSCs cultured for 3 weeks in osteogenic c, e and adipogenic conditions d, f. Magnification 20×; scale bar, 100 μm. (PDF 1843 kb) [file 13287_2015_166_MOESM4_ESM.pdf]
